# Developmental pathways for social understanding: linking social cognition to social contexts

**DOI:** 10.3389/fpsyg.2015.00719

**Published:** 2015-05-29

**Authors:** Kimberly A. Brink, Jonathan D. Lane, Henry M. Wellman

**Affiliations:** ^1^Department of Psychology, University of Michigan, Ann Arbor, MI, USA; ^2^Peabody College of Education and Human Development, Vanderbilt University, Nashville, TN, USA

**Keywords:** infancy, social cognition, theory of mind, continuity, longitudinal predictions

## Abstract

Contemporary research, often with looking-time tasks, reveals that infants possess foundational understandings of their social worlds. However, few studies have examined how these early social cognitions relate to the child’s social interactions and behavior in early development. Does an early understanding of the social world relate to how an infant interacts with his or her parents? Do early social interactions along with social-cognitive understandings in infancy predict later preschool social competencies? In the current paper, we propose a theory in which children’s later social behaviors and their understanding of the social world depend on the integration of early social understanding and experiences in infancy. We review several of our studies, as well as other research, that directly examine the pathways between these competencies to support a hypothesized network of relations between social-cognitive development and social-interactive behaviors in the development from infancy to childhood. In total, these findings reveal differences in infant social competences that both track the developmental trajectory of infants’ understanding of people over the first years of life and provide external validation for the large body of social-cognitive findings emerging from laboratory looking-time paradigms.

## Introduction

Human infants live in a social world and they develop expectations and understandings about people’s actions and interactions in that world. Hypothetically, their understandings—their social cognitions—simultaneously shape and are shaped by their social lives and interactions. Moreover, early infant social understanding and interactions should hypothetically shape later social cognition and social behavior in preschool, and beyond.

**FIGURE 1:**
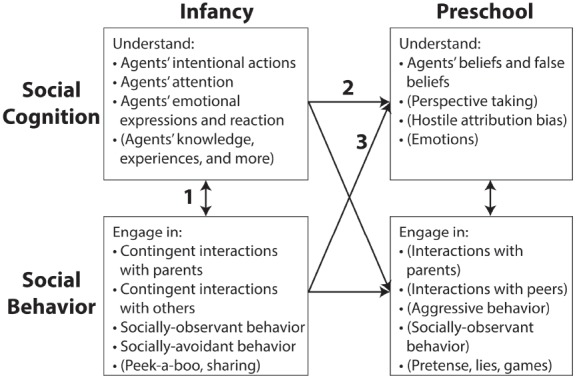
**Hypothesized theoretical framework for comprehensively looking at the pathways between early childhood social cognitions and social behavior.** Path 1 is examined in Study 1. Path 2 is examined in isolation in Study 2. Paths 2 and 3 are both assessed in Study 3. Social cognitions and behaviors not explicitly dealt with in this paper appear in parentheses (topics listed are for illustrative purposes and are not exhaustive). Social cognitions and social behaviors outside of parentheses are those we directly address in this paper.

Figure 1 outlines a theoretical framework for thinking about these developmental transactions, pathways, and achievements. Much is known about several of the topics within each box—e.g., infants’ understanding of intentional action, preschoolers’ understanding of false belief, and the nature of various parent-child interactions in infancy. The connections between these boxes, however, are only just beginning to be examined. Thus, considering this framework as a guide, our current knowledge is patchy and incomplete. In this article, we aim to help fill in this bigger theoretical picture about how early social cognition is informed by social context and *vice versa*. First, we review and report three illustrative studies of our own that tackle the ways in which early social cognition and social behavior fit together. These studies focus on the links labeled as 1, 2, and 3 in Figure 1. Using those studies as anchors, we review other emerging research that addresses these same links. Finally, we briefly discuss portions of this network that still need to be studied.

## Study 1: Relations between Intention Understanding and Infants’ Larger Social Experiences

### Background

Two methodological approaches characterize contemporary research on infant development: looking-time methods that reveal infants’ basic understandings of the world, and measures of individual differences that characterize variation among infants in their social contexts (e.g., parent–infant interaction or infant temperament). Surprisingly little research combines both of these methods and perspectives and this is particularly true in research encompassing infant social cognition.

Based on numerous recent studies of infant social cognition, it is now well accepted that during their first year of life infants come to understand that intentional, or goal-directed, states underlie the actions and expressions of others (Baird and Astington, 2005; Sommerville et al., 2005; Akhtar and Martinez-Sussmann, 2007; Tomasello et al., 2005). As noted in Figure 1, this includes appreciating agents as intentional actors (engaged in deliberate acts like reaching for and getting things) and as intentional experiencers (experiencing states like desires for, emotions about, and perceptions of things). Although these characterizations rest partly on studies where infants engage in active interaction with others (Carpenter et al., 1998; Saylor et al., 2007; Gräfenhain et al., 2009), the large majority of the relevant studies have used looking-time paradigms where infants look at and react to agents’ actions and expressions (e.g., Gergely et al., 1995; Woodward, 1998; Sodian and Thoermer, 2004).

At the same time, much research in the attachment and temperament traditions has examined infant and parent–infant social actions and interactions. Specifically, attachment research has shown that the sensitivity of mothers’ responses to their infants (Rosenblum et al., 2008; Bigelow et al., 2010) is consistently associated with a secure attachment style. Also, infant attachment status predicts and is predicted by aspects of mother–infant responsiveness to each other’s actions, communications, and mother–infant affect attunement (e.g., Beebe et al., 2010). These findings suggest that the quality of mother–infant interaction could also lead to enhanced social-cognitive understanding of the sort measured in infant laboratory assessments. Sensitive, responsive, contingent mother–child interactions might sensitize infants to the intentions and desires that underpin such behaviors. Beyond attachment research, infant temperament additionally seems a good candidate to consider because it is widely argued to reflect important individual differences in infancy that further influence infants’ experiences and interactions within their social worlds.

Although infant looking-time studies have provided the foundation for many theories of social-cognitive development, these studies rarely consider individual differences (as opposed to age-related differences) in infants’ understandings. Thus, they also rarely include analyses of infants’ social context, temperament, and parent–infant interaction, and so almost never examine the relations between infants’ social experiences or social behavior and their laboratory-based social cognitions. This seems like an important task in its own right and, moreover, such integrative research would also shed light on the ecological validity of the paradigms and findings so often used to study infant social cognition in the laboratory.

In a recent study (Dunphy-Lelii et al., 2014), therefore, we reported on two tasks implemented concurrently when infants were about 12 months old: (1) a traditional looking-time paradigm in which infants witness intentional action-emotion displays, and (2) caregiver–infant interaction episodes. This allowed us to examine relations between social cognition, via looking-time intention-understandings, and social behavior (pathway 1 in Figure 1) by taking advantage of individual differences in both. Thus, this first study had the goal of providing insight into relations between social behavior and social cognition (as well as traditional looking-time paradigms and measurements of social interaction) by investigating links between infant habituation to intentional-action displays and their social interactions and social temperaments.

### Overview of Study 1

For a looking-time paradigm we used one first reported by Phillips et al. (2002). In habituation trials infants saw a person look at one of two objects with an expression of interest and joy. Then they saw two test events in which the person either reached for the “liked” object (consistent event) or the not-liked object (inconsistent event). By 12–14 months of age most infants (80%) looked longer at the inconsistent test event, a finding consistent with other research demonstrating infants’ developing understanding of intentional agents. We chose this task because (a) it is representative of many used to assess intention understanding (e.g., Gergely et al., 1995; Woodward, 1998; Brandone and Wellman, 2009), (b) it involves infant appreciation of both actions (reaching) and emotions (liking), and (c) this same task captures reasonable individual differences in infants’ looking-time as shown in a prior study (Wellman et al., 2004).

To assess infant and mother–infant social interaction differences we included measures of the sort often used to examine the quality of mother–infant interaction in attachment and temperament research. In particular, by observing mother–infant interactions, we assessed the quality of the interaction and the infant’s temperamental orientation to and away from other people.

Almost one hundred 10- to 12-month-old infants were seen in a multi-phase research session lasting 40–50 min. After warm up, the protocol began with a looking-time procedure using the paradigm from Phillips et al. (2002) just described. Caregiver and infant then adjourned to a large playroom housing several toys and furniture where they participated in a 16-min session consisting of free play and novel object interaction (95% of the caregivers were mothers, so we will refer to our data as mother–infant interaction).

#### Social Cognition via Looking-time Measures

Looking-time tasks typically have two phases: a habituation or familiarization phase and a test phase. In social-cognitive research, during infant-controlled habituation, infants view an agent’s (or agents’) intentional behavior and emotional expressions over multiple trials until they become habituated—for example the agent looks at object-A rather than object-B with interest and pleasure. Then infants see test events that probe their understanding of the original actions—for example, the agent then reaches for object-A (a consistent test event, consistent with the agent liking and wanting that object) or object-B (an inconsistent test event). In this study we focused on individual differences in infants’ decrement (or, conversely, maintenance) of attention during habituation to our emotion–action displays. Attention during habituation acts as a measure of infants’ ability to parse displays meaningfully and their interest in those displays; that is, differences on this measure represent differences in processing or interest as infants come to a stable impression of the displayed action as being intentional. Infants’ attention to intentional-action displays during habituation, as measured by decrement of attention, has been especially revealing in prior research (Wellman et al., 2004; Aschersleben et al., 2008). Our measure (see also, Wellman et al., 2004) essentially involved subtracting infant looking on the last trials of habituation from their looking on the first trials (and then dividing by infant total habituation looking, as recommended by Bornstein and Sigman, 1986). So a higher score means infants’ attention decreased sizably and quickly whereas a lower score means infants sustained their attention longer and at higher levels.

We do not focus here on infant differential looking to consistent vs. inconsistent test events because in past social-cognitive research (Wellman et al., 2004; Aschersleben et al., 2008), it has often proven less revealing as an individual-differences measure. More focally, test event looking yielded little of significance in our research as well.

#### Social Interaction

Measures of mother–infant free play behavior could be collected by coding highly quantified observational tallies of specific acts (numbers of infant reaches, or points, or mother looks at infant per minute). But, more typically research has coded global, aggregated categories (Hofer et al., 2008; Slaughter et al., 2008; Kaitz et al., 2010). Arguably, global aggregates capture important individual differences at a more informative level of analysis (Sroufe and Waters, 1977) and we concentrated on those. Using such aggregates, we focused on four key constructs inspired by findings from attachment, temperament, and social-interaction research: quality of mother–infant interaction, socially observant temperament, joint attention, and imitation.

**FIGURE 2:**
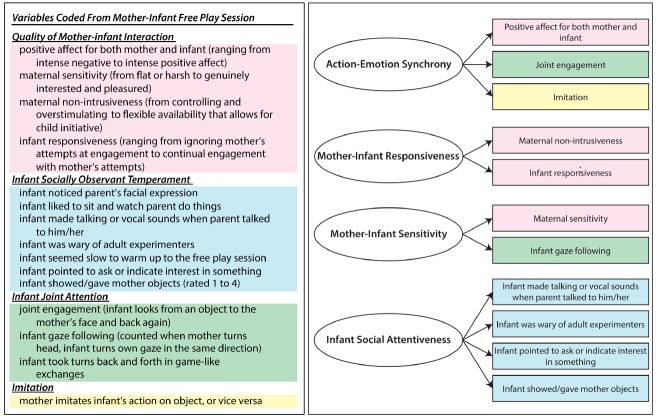
**Two separate sets of aggregates examined in Study 1.** The set on the left is organized into four *a priori* aggregates and color-coded. The set on the right is organized into four aggregates that were validated empirically within the study. Specific items from the *a priori* aggregates on the left contributed to the empirically validated aggregates, as indicated by the color of those items, on the right.

Because the quality of mother–infant interaction leads to enhanced attachment, it might additionally lead to enhanced social-cognitive understanding of the sort measured in infant laboratory assessments as well; infants who interact contingently with others who are sensitive to their own states and actions would be well situated to notice intentional actions and interactions and thus to achieve a more advanced understanding of them. In our data, *quality of mother–infant interaction* was based on aggregating four sets of global ratings from the free play observations as outlined on the left in Figure 2.

We also examined infants’ *socially observant temperament* (i.e., an infant’s attentiveness to social phenomena). Some aspects of temperament (e.g., activity level) describe infants’ motoric proclivities, but others (e.g., social reactivity or avoidance) are more related to infants’ social tendencies. Because these latter aspects of temperament can influence a child’s experiences and interactions within their social world, they could also impact early social-cognitive understanding. Indeed Wellman et al. (2011) showed that a “socially observant” temperament in early preschool longitudinally predicts enhanced theory of mind 2 years later (see also Lane et al., 2013). This temperament profile, however, was established in work with preschool children. Based on temperament items designed for infants and toddlers (Putnam et al., 2008), we devised items to assess something equivalent in infants. In general, socially observant infant temperament was coded based on whether infants noticed their parent’s facial expressions, liked to sit and watch their parents do things, and made talking or vocal sounds when parents talked to them.

Two other relevant social experiences arguably could play supporting roles in infants’ emerging understanding of others as intentional agents. *Joint attention* is a hallmark of early cognitive development in which infants begin to coordinate objects into their previously dyadic social interactions—they begin to monitor another’s attentional stance toward themselves and the object (see, e.g., Bakeman and Adamson, 1984). Infant *imitation* also has a long history of being studied as both a precursor to, and result of, increased social-cognitive competence. Deliberate infant imitation is evident as early as the second half of the first year (see Bauer and Kleinknecht, 2002), and it is related to both infant language development and mother–infant responsiveness (e.g., Masur and Olson, 2008).

### Findings

Many of the numerous aspects of infants’ and mothers’ behavior in our free-play/teaching scenarios that we coded are listed in Figure 2. In that figure, on the left behaviors are organized into *a priori* aggregates that we hypothesized might predict infants’ looking-times based on the four constructs we outlined earlier (i.e., the quality of mother–infant interaction, socially observant infant temperament, joint attention, and imitation). In initial analyses, we correlated these aggregates with differences in decrement of attention in the looking-time task. In Dunphy-Lelii et al. (2014) we reported similar relations without controlling for age, as all the participants were infants. But these infants ranged in age from 10 to 12 months and the age span between youngest and oldest infants was 10 weeks. This is sizable for infants aged, on average, 49 weeks, so in what we now report, all analyses are controlled for age in days at infant testing.

The four left-column aggregates describing the interactions between mother and child all correlated with our habituation measure when controlling for age: quality of mother–infant interaction, *r*(69) = 0.31, *p* < 0.01; socially observant infant temperament, *r*(85) = 0.25, *p* < 0.05; imitation, *r*(72) = 0.27, *p* < 0.05; and joint attention, *r*(70) = 0.24, *p* < 0.05. Not shown in Figure 2, we also coded for infant attentiveness to objects (rather than persons) as an infant behavior that could provide discriminant validity because we predicted it would not correlate with social-cognitive understandings. As expected, that score did not correlate with our habituation measure either initially, *r*(86) = –0.03, *p* = 0.77 or when controlling for age, *r*(85) = –0.04, *p* = 0.75.

Equally important, however, was a more comprehensive empirical analysis of the interactions and behaviors that predict looking-time measures of infant intention understanding. Here we began with the individual items listed on the left in Figure 2, but considered their internal organization further. Beginning with factor analysis and then adjusting and deleting items to achieve factors with good internal consistencies, we arrived at the four factors on the right in Figure 2. Items such as *infant and maternal affect* and *joint engagement* summed into a highly consistent factor (Factor 1), which we labeled as “action–emotion synchrony.” *Infant responsiveness* and *parental non-intrusiveness* loaded highly onto Factor 2, which we called, “mother–infant responsiveness.” The items *maternal sensitivity* and *infant gaze following* loaded highly onto Factor 3, which we labeled “mother–infant sensitivity.” And finally, several additional items captured a social-temperament factor that we called “infant social attentiveness” (Factor 4).

We entered these four derived factors—those on the right in Figure 2—into a regression predicting our primary habituation measure. To test the possibility that something like general object-centered attentiveness or general cognitive “maturity” would account for our findings, we entered the object-attentiveness measure noted earlier as well as age into this regression predicting our primary habituation measure; this step was not significant. We then entered our four key social factors, and those factors accounted for an additional 17% of variance in our habituation measure, *R*^2^_change_ = 0.17, *F*_change_ (4,63) = 3.31, *p* < 0.05. In this model, the infant social attentiveness aggregate (β = 0.26, *t* = 2.01, *p* < 0.05) and mother–infant responsiveness aggregate (β = 0.25, *t* = 2.01, *p* < 0.05) were independent predictors of social attention during the habituation portion of the looking-time study.

### Discussion

These findings indicate that infant social cognition, as demonstrated by performance in infant looking-time procedures, does relate to infants’ social interactive behavior. More specifically, individual differences in how infants parse and habituate to intentional action displays within laboratory looking-time research clearly relates to mother–infant interaction patterns, in particular the quality of mother–infant interaction. Additionally, infant looking-time performance also relates to infants’ social temperament, in particular, their disposition for attending to another person’s social behaviors (e.g., facial expressions and speech). These relations between looking-time performance and aspects of infant social experience provide validity to the assertion that common laboratory assessments of social-cognitive understanding do, in fact, tap important formative social understandings in infants.

Fortunately, our study does not stand alone; a small set of other research has tackled similar issues (generally pathway 1 in Figure 1). Brune and Woodward (2007) reported that 10-month-olds’ looking-time responses to a display linking a person’s gaze to an intended object was related to their engagement in joint attention during a mother–infant play session. Hofer et al. (2008) reported that 6-month-olds whose mothers were rated to have a modestly controlling interaction style encoded actions in terms of the agent’s goals, while those whose mothers were rated as sensitive or unresponsive did not. In an integration of looking-time and attachment research, Johnson et al. (2007) showed that securely attached infants (but not insecurely attached ones) looked significantly longer at an animated display in which a large “mother” object appeared to intentionally abandon a smaller “baby” object.

More recently, Licata et al. (2014) reported a study that partly parallels ours, but with 7-month-olds. These authors had 37 infants participate in Woodward’s intention-understanding looking-time task (e.g., Woodward, 1998) and then videotaped mother–infant interaction in a 10-min free play episode. They assessed mother–infant interaction by using Biringen’s (2000, 2008) Emotional Availability Scale, which involves global ratings of six dimensions such as maternal-sensitivity, maternal non-intrusiveness, and child-responsiveness. These dimensions were highly intercorrelated, so in a regression analysis controlling for age and infant activity level they entered only a general emotion-availability (EA) aggregate. EA was a significant predictor of children’s looking-time performances. Licata et al. (2014) concluded that EA captured general mother–infant interaction quality; thus combining their findings with ours, the quality of mother–infant interaction significantly relates to infant intention understanding as measured in looking-time tasks for 7-, 10-, 11-, and 12-month-olds. Interestingly, both we (Dunphy-Lelii et al., 2014) and Licata et al. (2014) also collected measures of maternal-mind-mindedness (Meins et al., 2003) from the free-play interactions, and in neither study did mind-mindedness predict infants’ looking-time intention understanding.

Because this first study (as well as the ones by Brune and Woodward, 2007; Licata et al., 2014) was concurrent, we could reveal relations between the laboratory and semi-naturalistic social experiences, but could not determine the direction of these relations: Infant social-interactive experiences and proclivities could contribute to enhanced intention understanding and *vice versa*. Indeed, as we hypothesize in pathway 1 of Figure 1, most likely the relationship is bi-directional and transactional.

## Study 2: Looking-Time Differences Predict 4-Year-Old Theory of Mind

In Study 2, we examined whether looking-time measures in infancy predict later social-cognitive understanding. Beginning with a study by Wellman et al. (2004), several studies have now examined this link—pathway 2 in Figure 1. But the current study, an updated version of Wellman et al. (2008), provides the most extensive and controlled evidence that we are aware of for a pathway from infants’ laboratory assessed social cognition to later preschool theory of mind. For measures of infant social cognition, we examined looking-time behaviors on the habituation task used in Study 1. For preschool theory of mind, we measured false-belief understandings.

### Overview of Study 2

Forty-five of the 10- to 12-month-old infants that participated in the looking-time habituation task in Study 1 returned to the laboratory at approximately 4 years of age to participate in a series of cognitive assessments. Focally these children were assessed on their theory of mind.

We also assessed children’s IQ, language competence (vocabulary), and executive functioning at 4-years because theory of mind has been linked to maturity of general information processing abilities. Moreover, for 40 years, it has been clear that infant attention to perceptual-object displays (such as familiar vs. novel objects and images) in looking-time studies predicts later IQ (Bornstein and Sigman, 1986; McCall and Carriger, 1993). These perceptual-attention findings are consistently interpreted as demonstrating developmental continuity in general information processing, such as memory-encoding or executive function. Conceivably, correlations between infant intention-understanding and preschool theory of mind might represent just another example of continuity in such general cognitive processing, as opposed to continuity more specific to a domain of social cognition. Thus, we included measures to account for this possibility.

#### Social-Cognitive Measures

Study 2 utilized the same infant looking-time measure described in Study 1—decrement of attention during habituation to intentional-action displays. Children’s preschool theory of mind was assessed with several measures. Importantly, children completed two explicit false-belief tasks because these provide the most often used, standard assessment for preschool theory of mind (Wellman et al., 2001): a standard, contents false-belief task (from Wellman and Liu’s (2004), theory-of-mind scale) and a standard, change-of-locations task (a Sally–Anne task of the type first used by Baron-Cohen et al., 1985). The scores from these two tasks were summed to form a false-belief composite.

#### IQ and Executive Function Measures

For a brief IQ assessment we used two subscales of the Wechsler Preschool and Primary Scale of Intelligence (WPPSI; Wechsler, 1989) Vocabulary (a measure of verbal aptitude) and Block Design (a non-verbal measure tapping various capacities, including spatial understanding and logic).

Executive functions encompass several constructs (e.g., Zelazo et al., 1997), but, of these constructs, inhibitory control yields the strongest relations to theory of mind (Carlson and Moses, 2001). Inhibition also deserves attention because it has been posited to explain the continuity of IQ from infancy to childhood (McCall, 1994; McCall and Mash, 1995). We used two common assessments of preschool inhibitory control—Whisper and Bear/Dragon (Kochanska et al., 1996; Carlson and Moses, 2001). For example, in the whisper task, children see 10 cartoon characters, some well-known (e.g., Winnie the Pooh, Elmo) and some not (e.g., Marvin the Martian, Petunia the Pig) and are asked to whisper the names of the characters that they know. Children are inclined to blurt loudly the names of the characters they know, and thus whispers reflect more sophisticated inhibitory control and earn children higher scores on this task.

### Findings

Focally, children’s scores as infants in the looking-time task were correlated with their preschool theory of mind. Children’s looking-time performance during infancy negatively correlated with the false belief composite when controlling for age, *r*(42) = –0.38, *p* = 0.01. That is, infants who sustained interest in (and therefore had smaller decrements in attention to) human intentional action displays during habituation at 1 year had enhanced theory of mind at age 4-years as indexed by better performance on the false belief tasks. Conceivably this association might be fully explained by verbal competence, performance competence (or a combination of the two representing overall IQ), and/or executive function. However, when we simultaneously controlled for age at infant testing, WPPSI Vocabulary and Block Design, and both executive function tasks, the relation between infant decrement of attention and preschool false-belief understanding remained significant, *r*(35) = –0.38, *p* < 0.05.

### Discussion

This second study demonstrated that, not only are individual differences in infant looking-time performance to intentional action displays predicted by parent–infant social interactive experiences, those looking-time differences also predict later theory of mind at 4 years of age. Moreover, just as for Study 1, our data are not the only relevant findings.

Specifically, Wellman et al. (2004) initially found a correlation between infant social attention and 4-year-olds’ performance on a false-belief composite for eighteen 14-month-olds. Replicating Wellman et al. (2004), Aschersleben et al. (2008) found a similar correlation between infant attention to goal-directed action and 4-year-old false-belief understanding for 20 German 6-months-olds. Yamaguchi et al. (2009) also reported a similar result: 4-month-old attention to goal-directed action (in a simpler procedure using animated circles and triangles) predicted later theory of mind (false-belief understanding) in a group of 17 U.S. children. Moreover, in a parallel study of fifteen 4-month-old infants, attention to *physical* stimuli (i.e., discrimination of tones rather than intentional-action stimuli) did not predict later theory of mind at 4 years (Yamaguchi et al., 2009). Notably, the 45 infants in our Study 2 more than doubled the samples used in these other studies and yielded a substantial correlation between later false-belief understanding and 10- to 12-month olds’ social attention.

Across several of these studies, the relation between infant social attention and later theory of mind is consistent and robust in the sense of remaining essentially undiminished when measures of more general cognitive processes are controlled. Wellman et al. (2004) used a single measure of verbal IQ (Peabody Picture Vocabulary Test); Aschersleben et al. (2008) used a single composite measure of language competence (the SETK; Grimm, 2001). And, as just noted, Yamaguchi et al. (2009) showed that one measure of attention to physical displays failed to predict later theory of mind. Of course, any single control is limited. For this reason, we examined multiple measures of general cognitive processing including verbal and non-verbal measures of general IQ and measures of executive functioning. More generally, verbal competence, general IQ, and executive function are complementary aspects of general information processing that make substantial and independent contributions to preschool theory-of-mind performance. Our Study-2 findings demonstrate continuity from infant social attention to preschool theory of mind with all three factors measured and controlled.

This social-cognitive continuity is consistently evident for measures of attention during habituation, but within the same studies (Wellman et al., 2004, 2008; Aschersleben et al., 2008), it does not appear for measures of test event looking (but see, Yamaguchi et al., 2009). We return to this issue in the General Discussion. Regardless, social cognition evidences distinctive infant-to-preschool continuities indicating that theory of mind constitutes its own domain of cognitive development. Infant social-cognitive understanding is not only early achieved as revealed in sensitive looking-time paradigms, it is formative for further developmental advances in theory of mind as hypothesized in pathway 2 of Figure 1.

## Study 3: Using Infant Individual Differences to Predict Preschool Social Cognition

While important, infant looking-time measures do not capture all of infant social cognitions or all of the predictive variance between infant social competence and experience and later social cognition. Indeed, Study 1 shows that the infant looking-time data and social-interactive measures interrelate. One possibility, therefore, is that looking-time measures are essentially a proxy for early infant–mother social experiences that themselves influence later social cognition including theory of mind. Or, more in line with the pathways outlined in Figure 1, it is possible that both social-cognitive competence (indexed in looking-time studies) and social-interactive behavior and temperament might independently contribute to the further development of preschool social cognition. If that is the case, then we would want to know the extent to which both types of measures utilized in concert predict preschool social-cognitive outcomes. We tackle these issues in Study 3.

In this final study we take advantage of the fact that the children for whom we have longitudinal data in Study 2 were also in Study 1. Thus we can combine information about social differences in the parent–infant interaction measures focal to Study 1, plus looking-time differences focal to Studies 1 and 2, as well as theory-of-mind data from the same children at 4 years of age. This allowed us to address several crucial questions: What features of infant social-cognitive and social-interaction experiences combine to best predict later theory of mind? What factors are, separately, the most influential? And what is the total predictive power of these factors?

### Overview of Study 3

Forty-three children participated in a series of social interaction and social-cognitive assessments: mother–infant interactions and looking-time habituation tasks at 10–12 months of age, as well as theory of mind tasks at 4 years. Social-interaction measures, looking-time measures, and preschool theory of mind measures were those described in Studies 1 and 2.

### Findings

We used regression modeling to determine the independent and joint contributions of infant social-interactive measures (i.e., the four measures: quality of mother–infant interaction, socially observant infant temperament, infant joint attention, and imitation) and habituation scores to children’s theory of mind at age 4. Given the number of measures, not all participants had complete data. Specifically, seven participants were missing data at random; therefore, five iterations of imputation were performed in order to predict the values of the missing data. The participants that were missing data did not differ systematically from the other children in terms of demographics or the other variables of interest (quality of mother–infant interaction, socially observant temperament, joint attention, or imitation). In all five iterations the exact same patterns of significance and non-significance emerged, therefore we report the results based on the pooled values of the five imputations.

Again, to test the possibility that something like general object-centered attentiveness or general “maturity” would account for our findings, we first entered the infant object-attentiveness measure as well as age into a regression predicting false-belief understanding at age 4; this was not significant, *R*^2^ = 0.07, *F*(2,38) = 0.27, *p* = 0.47. Entering our four infant social-interactive measures combined with habituation scores in a second separate analysis did significantly predict children’s theory of mind at age 4, *R*^2^ = 0.27, *F*(5,37) = 2.79, *p* < 0.05. Of the five measures, the infant habituation measure (*t* = 2.83, *p* < 0.01) and socially observant temperament (*t* = 2.34, *p* < 0.05) independently significantly predicted performance on the false-belief tasks.

Similarly, using stepwise regression, the model including the four infant social-interactive aggregates and the habituation measure was reduced to two independent and significant predictors of preschool false belief understanding: socially observant infant temperament and the infant habituation measure. Socially observant infant temperament and infant performance on the looking-time task predicted 26% of the variability in preschool false belief understanding, *R*^2^ = 0.26, *F*(2,40) = 2.87, *p* < 0.05.

### Discussion

Study 3 demonstrates that both infant performance differences in looking-time paradigms and parent–infant interaction differences, and especially infant social temperament differences, independently predict later theory-of-mind performance. Neither is a mere proxy for the other, and together infant individual differences of both sorts more powerfully predict preschool social cognition. Indeed, when our several predictive factors were entered jointly, the overall regression model accounted for a sizable amount of variance in preschool theory of mind, and two variables alone—one looking-time measure and one social-interaction measure—accounted for 26% of the variability in 4-year-olds’ false belief performance.

Pathways from infant social-interaction experiences to later social cognition (pathway 3 in Figure 1) are not well studied, but, still, our Study-3 findings are complemented by several others. Nelson et al. (2008) examined 30 min of mother–infant interaction for children aged 18–21 months. From that they extracted several measures of joint attention or joint engagement and related these to children’s false-belief competence as 4-year-olds. Two measures were particularly predictive: *Coordinated joint engagement* (where infant as well as mother managed the dyad’s joint attention to events) and *symbol-infused joint engagement* (where child participation in joint events included verbal reference to both the mother and to the events). Higher amounts of coordinated and symbol-infused joint engagement in these toddlers significantly predicted better false-belief performance when the children were 4 years of age, and did so even after language competence was controlled. Likewise, in an early study of a small sample of 13 infants, joint attention at 20 months (measured as gaze switching between an adult and a salient toy in a laboratory task) was predictive of later preschool-age theory of mind, and remained significant after IQ and language competence were partialed out (Charman et al., 2000).

Other measures of infant social-cognitive understanding could also be related to later preschool theory-of-mind performance. Recently, investigators of infant social cognition have expanded beyond examination of infants’ understanding of intentional action and emotion to focus on infants’ implicit understanding of agents’ knowledge and beliefs as well (e.g., Onishi and Baillargeon, 2005; Southgate et al., 2007; Buttelmann et al., 2009). Implicit understanding of false belief is assessed via violation of expectation looking-time studies as well as anticipatory looking measured via eye-tracking methods. Thoermer et al. (2012) have shown that belief-based anticipatory looking measures at 18 months of age longitudinally predict false-belief reasoning on standard verbal preschool tasks at 48 months.

Note that our study goes beyond such prior research in including younger infants and in including both social-interactive *and* looking-time measures. To reiterate, together infant individual differences of both sorts more powerfully predicted preschool social cognition.

## General Discussion

Returning to Figure 1, contemporary research on early childhood understanding of the social world should ideally encompass numerous constructs studied in the lab and in social interactions and, crucially, the transactional and longitudinal pathways that form and contextualize such understandings. While the field has, for the most part, explored bits and pieces of this developmental system separately, the appropriate combination of constructs, approaches, and ages can provide a clearer picture of the developmental trajectory of children’s social-cognitive understanding.

In this paper, we reviewed evidence from three studies conducted by our research group that examined pathways 1, 2, and 3 in our theoretical framework. We demonstrated that infants’ social-cognitive understanding, as measured by laboratory performance on social-cognitive tasks, was significantly associated with individual differences in social-interactive experiences and infant social temperaments (pathway 1). We also demonstrated that both infant social-cognitive understanding and infant social-interactive experiences and temperaments contributed independently to later social-cognitive competencies, specifically preschool theory of mind (pathways 2 and 3). And they do so even after age, IQ, language competence, and executive function are controlled.

It seems of special note that among our predictors, socially observant infant temperament was an important predictor of infant social cognition in looking-time tests, as well as a particularly important predictor of later, preschool social cognition. This finding takes its place beside an emerging set of findings concerning relations between preschool temperament and theory of mind achievements—preschoolers who are shy, socially observant, and non-reactive outperform their peers on false-belief tasks (Wellman et al., 2011; Lane et al., 2013). What we add to these findings are data from infants and that infants’ socially-observant temperament relates to their social cognition both concurrently (at 1 year) but also longitudinally, during the preschool years. Importantly, Mink et al. (2014) have also recently reported that infants with a shy temperament at 18-months exhibit a more advanced theory of mind by the early preschool years.

Note that this research showing early links between socially observant temperament and theory of mind spans several countries and cultures: the U.S. (the current studies; Wellman et al., 2011), China (Lane et al., 2013), and Germany (Mink et al., 2014). This is an impressive beginning, but of course, further research with other samples and especially with infants would be welcome. More generally, it will be important to determine the extent to which these relations between infant understanding of intentional agents, their social-interactive experiences, and early social-cognitive developments exist for other realms of social-cognition, not just theory of mind, but, for example, in infants’ and preschoolers’ moral intuitions.

We hasten to emphasize that this social attentiveness—reflected in both infant socially observant temperament as well as maintenance of interest in intentional action displays in our looking-time paradigms—should not be thought of as a purely dispositional factor inherent to the infant. In Study 1 social attentiveness, as measured by infant “temperament” ratings, was related to measures of mother–infant interaction and interaction quality. In Licata et al. (2014) attention to intentional action in their looking-time task was related to maternal emotional availability. In short, enhanced attention to social actors and social interactions in infancy powerfully impacts early childhood social cognition, but that enhanced social attention can be due to the efforts of the infant, the infant’s social partners, or mostly likely to both in concert. Determining the nature and social shaping of social attentiveness in early life is probably the single topic now most worth increased research efforts.

There are other pathways apparent in Figure 1 that we have not touched on here. For example, it is now well established that preschool theory of mind impacts preschoolers’ social behavior, such as the skilled interactions with and hence popularity with their peers (e.g., Watson et al., 1999; Diesendruck and Ben-Eliyahu, 2006), their ability to tell lies (e.g., Lee, 2013), their attempts to persuade others (e.g., Bartsch and London, 2000) and their engagement in games like hide-and seek (Peskin and Ardino, 2003). And preschool social behaviors, such as engaging in pretend play (e.g., Jenkins and Astington, 2000), engaging in family conversation about mind and emotion (e.g., Ruffman et al., 2002), and living in a family full of siblings and extended family members (e.g., Peterson, 2000), enhance preschool theory of mind. However, in keeping with a focus that includes infants, the remaining pathway of most import is the diagonal from infant social cognition to preschool social behavior. We know of no research that has tackled this pathway; it now seems of special import for future research.

More specific aspects of our findings also deserve mention. First, *both* more and less sustained attention to intentional action displays relate to infants’ interactions and later competences. Both of these relations make sense: Steeper decrement of attention in habituation can reflect coming more quickly to a consolidated and intentional understanding of action. It can index infants’ ability to parse intentional action displays meaningfully and thus habituate to them. Hypothetically, infants who more quickly habituate to complex displays of the sort that we present could be more practiced at understanding intentional action regularities and thus more quickly become habituated to them. That sort of relation may very well underpin the positive relation between decrement of attention and parent–infant interaction measures we found in Study 1.

At the same time, more sustained attention–and hence coming more slowly and less “steeply” to habituation criteria—could reflect greater interest in and deeper processing of intentional action. And such sustained attention to human intentional actions could promote later more advanced understanding. That sort of relation may very well underpin the negative relation we found between decrement of attention and later theory of mind in Study 2. Similarly, the relation between our infant temperament ratings and later theory of mind that we found in Study 3 seems to reflect the power of sustained, enhanced, infant social attentiveness.

Notably, this mix of significant relations, including both more quickly parsing intentional action and also greater attention to intentional action, occurs not only in our three studies but also throughout the literature. In Wellman et al. (2004) the relation between infant decrement of attention and later false-belief was positive, suggesting that by 14 months more quickly parsing intentional action predicts theory of mind at 4 years. Yet, to repeat, in Study 2 here (and thus in Wellman et al., 2008) the relation was negative. So for 10- and 12-month-olds, more sustained attention to intentional action displays predicted theory of mind at 4 years. Perhaps, then, for younger infants the key factor is sustained attention in habituation, but for older infants it is quicker, fluent intention-action processing. That cannot be the whole story, however, because in Aschersleben et al.’s (2008) study using a very different intention-action habituation display with 6-month-olds, it was infants who more quickly habituated—thus showing a larger attention decrement in habituation—that proved better at false-belief understanding as 4-year-olds. This abundance of differing relations requires closer scrutiny in future research.

Second, in our research and others (e.g., Aschersleben et al., 2008), it was infants’ attention to intentional-action displays during habituation trials and not test trials that was especially revealing. Thus, social-cognitive processing revealed during habituation has proved particularly important. Yet, Brune and Woodward (2007) and Licata et al. (2014) found that, given their looking-time task, infant differences in response to novelty were also significantly related to parent–infant social behavior. Notably, our data, in contrast to theirs, demonstrate important continuities between our infant measures and preschool social cognition. This is an important addition, but the full pattern of relations between social cognition and social interaction, and the full pattern of continuities from infant intentional understandings plus social interactions to later theory of mind is an important topic for further research.

To summarize, future research should continue to explore the theoretical framework that we present here. Our research, along with the work of others, provides a strong foundation supporting conclusions that infants’ social-cognitive understanding, their social-interactive experiences, and their social temperaments significantly inter-correlate and contribute to later social-cognitive competencies. However, several remaining portions of the framework deserve further exploration. First, we proposed a number of pathways that have not yet been evaluated in the literature. How does infant social cognition relate to later preschool social interactions and behaviors? Additionally, future research should continue to assess the nature of the pathways between laboratory-based assessments of social cognition and later social understanding that we have only just begun to explore. More evidence is required to understand how individual differences in sustained attention and decrement of attention relate to later social developments. Additionally, the influence of sensitivity to novelty and sensitivity to familiar social stimuli should also be assessed.

To conclude, in keeping with the focus of this special issue, we demonstrate the importance of individual differences in research on early social cognition. Our specific focus was on individual differences anchored in infancy, particularly differences in everyday mother–infant interaction as well as laboratory-based looking-time tasks. Our results demonstrate that both approaches generate informative measures of individual differences, and that moreover used together such measures can be especially compelling. Future research on children’s social development should not focus solely on laboratory-based habituation measures nor individual differences in social experiences, but the combination of the two. More generally, our studies, along with recent confirmatory findings from Licata et al. (2014), Mink et al. (2014), Brune and Woodward (2007) and the like, underscore that social-cognitive development is a constructive process based in social interaction and observation.

### Conflict of Interest Statement

The authors declare that the research was conducted in the absence of any commercial or financial relationships that could be construed as a potential conflict of interest.
